# Obstructive Sleep Apnea Is Associated with Liver Damage and Atherosclerosis in Patients with Non-Alcoholic Fatty Liver Disease

**DOI:** 10.1371/journal.pone.0142210

**Published:** 2015-12-16

**Authors:** Salvatore Petta, Oreste Marrone, Daniele Torres, Maria Buttacavoli, Calogero Cammà, Vito Di Marco, Anna Licata, Anna Lo Bue, Gaspare Parrinello, Antonio Pinto, Adriana Salvaggio, Antonino Tuttolomondo, Antonio Craxì, Maria Rosaria Bonsignore

**Affiliations:** 1 Sezione di Gastroenterologia e Epatologia, DiBiMIS, University of Palermo, Palermo, Italy; 2 National Research Council, Institute of Biomedicine and Molecular Immunology, Palermo, Italy; 3 Sezione di Medicina Interna, DiBiMIS, University of Palermo, Palermo, Italy; 4 Sezione di Malattie Cardio Respiratorie ed Endocrino Metaboliche, DiBiMIS, University of Palermo, Palermo, Italy; Institute of Medical Research A Lanari-IDIM, University of Buenos Aires-National Council of Scientific and Technological Research (CONICET), ARGENTINA

## Abstract

**Background/Aims:**

We assessed whether obstructive sleep apnea (OSA) and nocturnal hypoxemia are associated with severity of liver fibrosis and carotid atherosclerosis in patients with biopsy-proven NAFLD and low prevalence of morbid obesity. Secondary aim was to explore the association of OSA and hypoxemia with NASH and severity of liver pathological changes.

**Methods:**

Consecutive patients (n = 126) with chronically elevated ALT and NAFLD underwent STOP-BANG questionnaire to estimate OSA risk and ultrasonographic carotid assessment. In patients accepting to perform cardiorespiratory polygraphy (PG, n = 50), OSA was defined as an apnea/hypopnea index ≥5. A carotid atherosclerotic plaque was defined as a focal thickening >1.3 mm.

**Results:**

Prevalence of high OSA risk was similar in patients refusing or accepting PG (76% vs 68%, p = 0.17). Among those accepting PG, overall OSA prevalence was significantly higher in patients with F2-F4 fibrosis compared to those without (72% vs 44%; p = 0.04). Significant fibrosis was independently associated with mean nocturnal oxygen saturation (SaO_2_)<95% (OR 3.21, 95%C.I. 1.02–7.34; p = 0.04). Prevalence of OSA tended to be higher in patients with, than in those without, carotid plaques (64% vs 40%; p = 0.08). Carotid plaques were independently associated with %time at SaO2<90% >1 (OR 6.30, 95%C.I. 1.02–12.3; p = 0.01).

**Conclusions:**

In NAFLD patients with chronically elevated ALT at low prevalence of morbid obesity, OSA was highly prevalent and indexes of SaO_2_ resulted independently associated with severity of liver fibrosis and carotid atherosclerosis. These data suggest to consider sleep disordered breathing as a potential additional therapeutic target in severe NAFLD patients.

## Introduction

In a short time, the current epidemic of overweight and obesity may lead to an increase in prevalence of non-alcoholic fatty liver disease (NAFLD), a major cause of chronic liver disease worldwide [1–3]. A considerable proportion of patients with NAFLD is at risk of progression to cirrhosis [2,4], and development of cancer and cardiovascular events [5].

The pathogenesis of NAFLD is multifaceted and incompletely understood. In this complex landscape, a number of metabolic factors like obesity, insulin resistance (IR) and diabetes [6–8] have been identified as involved in the pathogenesis and progression of liver fibrosis. Notably, the same factors have been confirmed as predictors of cardiovascular events among patients with NAFLD. Besides, new actors in the pathogenesis of liver and cardiovascular damage in NAFLD are emerging, such as genetic background [9], fructose consumption [10], hyperuricemia [11], and vitamin D deficiency [12].

Obstructive sleep apnea (OSA) is characterized by upper airway collapse during sleep, intermittent hypoxia, sympathetic and inflammatory activation, sleep disruption, and increased cardiovascular risk [13]. A recent large scale Taiwanese population study identified OSA as an independent predictor of incident liver disease [14], and according to several studies OSA-associated risk could be particularly high for NAFLD [15–29]. Indeed, studies in humans and animal models consistently showed that chronic intermittent hypoxia, a common feature of OSA, is a potent pathogenic trigger not only for cardiovascular, but also for metabolic alterations. Along this line, some studies assessed the potential impact of OSA on liver damage in patients with NAFLD. Most of these studies on adult patients reported an association between OSA and ALT levels or histological severity of liver damage, but were performed in morbidly obese patients [15–23]. Similar data were reported in studies on children with NAFLD [25,26]. The only studies on non-morbidly obese adult NAFLD patients gave inconsistent results. However, these studies estimated OSA prevalence only by questionnaires [27,28], or they did not always obtain a histological diagnosis of NAFLD [24,27,29].

Finally, carotid atherosclerosis is frequently associated with OSA [30–32]. NAFLD patients are at high cardiovascular risk per se [33], but it is unknown whether nocturnal respiratory disorders may independently contribute to vascular damage in this high-risk population.

Having this in mind, the main goal of this study was to assess whether OSA, instrumentally diagnosed by respiratory polygraphy, and indexes of nocturnal oxygen saturation (SaO2) are associated with severity of liver fibrosis and carotid atherosclerosis in patients with biopsy-proven NAFLD and without morbid obesity. Secondary aim was to explore the association of OSA and indexes of oxygen saturation with nonalcoholic steatohepatitis (NASH), severity of steatosis, ballooning and lobular inflammation.

## Patients and Methods

### Patients

The study involved 126 consecutive patients with NAFLD, recruited at the Gastrointestinal & Liver Unit of Palermo University Hospital, and fulfilling all the inclusion and exclusion criteria detailed below. The main inclusion criterion was a histological diagnosis of NAFLD on a liver biopsy done less than 6 months before enrollment, showing steatosis (>5% of hepatocytes) with or without necroinflammation and/or fibrosis including cirrhosis. The pre-biopsy assessment of NAFLD was based on chronically elevated ALT for at least 6 months and alcohol consumption of <20 g/day in the previous year (also confirmed by a questionnaire). Exclusion criteria were: (1) decompensated cirrhosis (presence of jaundice, ascites or encephalopathy); (2) hepatocellular carcinoma; (3) liver disease of different or mixed etiology (excessive alcohol consumption, hepatitis C, hepatitis B, autoimmune liver disease, Wilson’s disease, hemochromatosis, α1-antitrypsin deficiency); (4) human immunodeficiency virus infection; (5) previous treatment with antiviral therapy, immunosuppressive drugs and/or regular use of steatosis-inducing drugs (steroid, amiodarone, tamoxifen, etc.), as assessed at interview; (6) history of heart disease (both coronary or cardiac disease); (7) active intravenous drug addiction; (8) body mass index (BMI) exceeding 45 kg/m^2^; (9) previous diagnosis of, or assessment for, OSA.

The study was carried out in accordance with the principles of the Helsinki Declaration and its appendices, and with local and national laws. Approval of the study was obtained from the AOUP Policlinico Paolo Giaccone of Palermo Review Board and Ethics Committee, and written informed consent was obtained from all patients.

### Clinical and Laboratory Assessment

Clinical and anthropometric data were collected at the time of liver biopsy. Patients were classified as normal weight (BMI 18.5–24.9 kg/m^2^), overweight (BMI 25–29.9), or obese (BMI ≥30). Waist circumference (WC) was measured at the midpoint between the lower border of the rib cage and the iliac crest. Visceral obesity was diagnosed when WC was ≥102 cm in males and ≥88 cm in females [34]. The diagnosis of arterial hypertension was based on the following criteria: systolic blood pressure ≥140 mm Hg and/or diastolic blood pressure ≥90 mm Hg (measured three times within 30 minutes, in the sitting position and using a brachial sphygmomanometer), or use of blood-pressure-lowering agents. The diagnosis of impaired glucose tolerance or type 2 diabetes was based on the revised criteria of the American Diabetes Association, using a value of fasting blood glucose ≥100 to <126, and ≥126 mg/dl, respectively [35]. In patients with a previous diagnosis of type 2 diabetes, current therapy with insulin or oral hypoglycemic agents was recorded.

A 12-hour overnight fasting blood sample was drawn at the time of biopsy to determine the serum levels of ALT, total cholesterol, LDL-cholesterol, HDL-cholesterol, triglycerides, plasma glucose, insulin, and platelet count. Insulin resistance (IR) was determined according to the homeostasis model assessment (HOMA) method [36], as: Insulin resistance (HOMA-IR) = Fasting insulin (μU/mL) x Fasting glucose (mmol/L)/22.5.

### Assessment of Liver Histology

Slides were coded and read by one pathologist, who was unaware of the patient’s identity and history. A minimum length of 15 mm of biopsy specimen or the presence of at least 10 complete portal tracts was required [37]. Steatosis was assessed as the percentage of hepatocytes containing fat droplets (minimum 5%), and evaluated as continuous variable. The Kleiner classification [38] was used to grade steatosis, lobular inflammation, and hepatocellular ballooning, and to stage fibrosis from 0 to 4. NASH was diagnosed when steatosis, ballooning and lobular inflammation of any grade were present at the same time.

### Obstructive Sleep Apnea assessment

Patients filled the validated STOP BANG [39] clinical questionnaire within 3 months from liver biopsy in order to identify subjects at high risk for OSA. The STOP BANG contains 8 questions: 1. Snoring: Do you snore loudly (loud enough to be heard through closed doors)?; 2. Tired: Do you often feel tired, fatigued, or sleepy during daytime?; 3. Observed: Has anyone observed you stop breathing during your sleep?; 4. Blood pressure: Do you have or are you being treated for high blood pressure?; 5. BMI: BMI more than 35 kg/m^2^?; 6. Age: Age over 50 yrs old?; 7. Neck circumference: >40 cm?; 8. Gender: Male?. A score higher than 2 indicates high risk for OSA.

Subjective sleepiness was evaluated by the Epworth Sleepiness Scale (ESS), and a score ≥10 was considered indicative of excessive daytime sleepiness [40].

Fifty out of 126 patients accepted to undergo a home nocturnal cardiorespiratory monitoring (Somnea, Compumedics, Abbotsford, Victoria, Australia). The following signals were recorded: thoracic and abdominal movements by inductance plethysmography, SaO_2_ by pulse oximetry, heart rate, nasal airflow by nasal pressure cannulae and pressure transducer, snoring, and body posture. Sleep period, considered as time between lights off in the evening and lights on in the morning, was identified based on patients’ diaries and on behaviour of the recorded signals. Signals recorded in the sleep period were manually analysed [41]. Apneas were scored when the airflow decreased by at least 90% from baseline for at least 10 seconds and classified as central, mixed or obstructive depending on occurrence of thoracoabdominal movements [41]. Hypopneas were scored when airflow decreased by at least 30% for ≥10 seconds and was associated with a SaO2 fall ≥4%. Apnea/hypopnea index (AHI) was calculated as the average number of apneas and hypopneas per hour of recording in the sleep period. An AHI ≥5 was used to diagnose OSA. SaO2 in the sleep period was automatically analysed and the following parameters were obtained, after manual elimination of possible artifacts: baseline SaO2 during wakefulness, mean SaO2, time spent with SaO2 <90% (T90), lowest nocturnal SaO2 value [41].

### Carotid artery evaluation

Carotid atherosclerosis was evaluated by an expert physician, blinded to the characteristics of patients, using a high-resolution B-mode ultrasonography equipped with a multifrequency linear probe.

Bilateral longitudinal projections at the level of the common carotid artery, of the bulb and of the internal carotid artery were taken. The intima-media thickness (IMT) was measured as the difference between the first (intima-lumen) and the second (media-adventitia) interface on the far wall of the common carotid arteries in a section free of plaques beginning 10 mm below their bifurcations and including the bifurcations for 10 mm. For each subject, three measurements on both sides were performed, i.e., the anterior, lateral, and posterior projection of the near and far wall. Maximum (outside the plaque) values of IMT were considered, and edge detection was performed manually. IMT measurements from the left and right side were averaged. A plaque was defined as a focal thickening >1.3 mm at one or more levels of carotid arteries or of their bifurcation [42].

### Statistics

Continuous variables were summarized as mean ± standard deviation or median (95% C.I.), and categorical variables as percentage. T-test or chi-square test were used when appropriate. Multiple logistic regression models were used to assess the factors independently associated with significant liver fibrosis and carotid plaques (main aims). Multiple logistic and ordinal regression models were used to assess the factors associated with NASH, severity of steatosis, ballooning and lobular inflammation. For SaO_2_ variables, the median was used as a cut-off value. Variables included in multivariate models were chosen as potential confounders, based on their significance at univariate analyses (p<0.10) and/or their biological plausibility.

High risk for OSA, age, gender and hypertension, as well as metabolic syndrome and its components were not included in the same models due to co-linearity.

Regression analyses were performed using SAS [43].

## Results

### Patients

We included 126 consecutive patients with NAFLD who responded to the STOP-BANG questionnaire, and underwent carotid ultrasonography and liver biopsy. At liver biopsy, approximately one patient in two had fibrosis ≥ 2 by Kleiner score, while two in three had diagnosis of NASH.

Seventy-six patients (60%) refused the sleep study, while 50 patients underwent cardiorespiratory polygraphy. S1 Table shows the comparison between NAFLD patients according to acceptance of the sleep study. The patients who accepted had a higher prevalence of metabolic comorbidities like obesity, hypertension and diabetes, even if prevalence of high risk for OSA assessed by STOP-BANG questionnaire was similar in the two groups, as well as the degree of liver damage in terms of steatosis, lobular inflammation, ballooning, NASH and fibrosis.

In patients who underwent sleep studies, mean age was 53 years, and there was a slight male predominance (58%). Most patients (60%) showed visceral obesity, 50% were hypertensive, and 40% were diabetic. Mean values for total, HDL-cholesterol, and triglycerides were within the normal range, whereas mean HOMA value was elevated (4.4 ± 2.4).

### Characteristics of patients with Obstructive Sleep Apnea (AHI≥5)

Demographic, clinical and metabolic characteristics of patient at low (n = 39) or high (n = 87) risk of OSA according to the STOP-BANG questionnaire are reported in S2 Table. Among patients who accepted the sleep study, an AHI***≥***5 was observed in 50% of the subjects, even if an AHI>30 (i.e. severe OSA) was found only in 20% of cases. Notably, all patients with AHI***≥***5 were in the high-risk group according to the STOP-BANG questionnaire, but the questionnaire correctly identified only 65% of the patients with OSA. Hence, sensitivity, specificity, positive predictive value and negative predictive value of STOP-BANG in diagnosing OSA in patients with NAFLD were 100%, 48%, 65% and 100%, respectively.

Characteristics of patients according to the presence or absence of AHI***≥***5 are reported in Table 1. As expected, patients with AHI***≥***5 were older and had a higher prevalence of obesity, hypertension and diabetes compared to those without OSA (p<0.05 for all). When considering oxygen saturation parameters, patients with AHI***≥***5 had worse mean SaO_2_ and T90 (p<0.01 for both). Notably, no differences in subjective sleepiness were observed between patients with and without OSA (Table 1) or between patients at low or high risk for OSA assessed by the Epworth questionnaire (S2 Table).

**Table 1:** Baseline Demographic, Laboratory, Metabolic, and Histological Features of 50 Italian Patients with biopsy-proven Non-alcoholic Fatty Liver Disease according to presence or absence of obstructive sleep apnea.

Variable	AHI<5	AHI≥5	P value
	n = 25	n = 25	
**Age–years**	49.6 ± 11.7	56.1 ± 9.1	0.03
**Male Gender—% of subjects**	56	60	0.77
**BMI—kg/m** ^**2**^	29.0 ± 3.6	33.5 ± 4.7	<0.001
**BMI<25 kg/m** ^**2**^ **- % of subjects**	12.0	4.0	
**BMI≥25-<30 kg/m** ^**2**^ **- % of subjects**	48.0	12.0	
**BMI≥30-<35 kg/m** ^**2**^ **- % of subjects**	32.0	40.0	
**BMI≥35-<40 kg/m** ^**2**^ **- % of subjects**	8.0	32.0	
**BMI≥40 kg/m** ^**2**^ **- % of subjects**	0	12.0	0.005
**Waist Circumference–cm**	101.6 ± 9.2	111.7 ± 11.4	0.001
**Visceral Obesity—% of subjects**	68	92	0.03
**Alanine Aminotransferase–IU/ml**	63.6 ± 34.5	66.5 ± 33.4	0.76
**Blood glucose–mg/dl**	97.0 ± 20.7	119.1 ± 38.8	0.01
**Insulin–IU**	17.1 ± 9.4	19.6 ± 11.6	0.49
**HOMA**	4.07 ± 2.17	4.78 ± 2.75	0.32
**Type 2 Diabetes- % of subjects**	20	60	0.004
**Arterial Hypertension- % of subjects**	20	80	<0.001
**Metabolic Syndrome—% of subjects**	20	24	0.73
**Smoking—% of subjects**	20	24	0.39
**HDL Cholesterol–mg/dl**	51.9 ± 12.1	56.8 ± 30.8	0.46
**LDL Cholesterol–mg/dl**	128.3 ± 33.0	107.0 ± 39.4	0.04
**Triglycerides–mg/dl**	124.3 ± 45.1	136.5 ± 86.6	0.53
**Intima Media Thickness—mm**	0.82 ± 0.18	0.83 ± 0.14	0.73
**Carotid Plaques—% of subjects**	40	64	0.08
**AHI–no/hour**	2 (0–4.8)	11 (5–52)	<0.001
**Basal SaO2 - %**	96.4 ± 0.9	95.6 ± 1.5	0.01
**Mean Sa02 - %**	94.8 ± 1.3	92.9 ± 2.2	0.001
**Mean Sa02 <95%—% of subjects**	28	84	<0.001
**T90 - %**	3.1 (0.3–6.3)	13.3 (6.5–42.7)	<0.001
**T90 >1%—% of subjects**	16	56	0.003
**ESS score**	5.5 ± 3.1	7.0 ± 3.3	0.11
**ESS score ≥10 (%)—% of subjects**	16	16	1.00
**Histology**			
**Lobular inflammation 2–3 - % of subjects**	44	43.4	0.97
**Steatosis grade 3 (>66%)—% of subjects**	20	34.7	0.25
**Ballooning—% of subjects**	64	91	0.02
**NASH—% of subjects**	60	88	0.02
**Fibrosis Stage 2–4 - % of subjects**	44	72	0.04

Abbreviations: IU, international units; HOMA, homeostasis model assessment; HDL, high density lipoprotein; LDL, low density lipoprotein; AHI, apnea-hypopnea index; SaO2, oxygen saturation; T90%, percentage of total sleep time spent with SaO2<90; ESS, Epworth Sleepiness Scale. Data are given as mean ± standard deviation, or as median (95% C.I.), or as %.

### The severity of liver damage is associated with high risk for OSA and lower oxygen saturation

In the entire cohort of 126 patients the prevalence of F2-F4 fibrosis was higher in patients at high risk compared to those without high risk for OSA (80.6% vs 57.8%, p = 0.006), this association remaining marginally significant after adjusting for confounders (O.R. 2.25, 95%C.I. 0.97–5.22; p = 0.05) (Table 2). Similar results were obtained when replacing WC and HOMA with metabolic syndrome in the model.

**Table 2:** Univariate and multivariate analyses of factors associated with F2-F4 Fibrosis in 126 patients with nonalcoholic fatty liver disease who were administered STOP-BANG.

Variable	Unadjusted Model	Adjusted Model
**Waist Circumference**	1.05 (1.01–1.09) 0.003	1.03 (0.99–1.07) 0.07
**HOMA**	1.38 (1.12–1.70) 0.002	1.23 (0.99–1.54) 0.06
**High Risk for OSA**	3.04 (1.36–6.78) 0.007	2.25 (0.97–5.22) 0.05

Abbreviations: HOMA, homeostasis model assessment; OSA, obstructive sleep apnea; AHI, apnea-hypopnea index; SaO2, oxygen saturation; T90, percentage of total sleep time spent with SaO2<90%.

**Fig 1:**
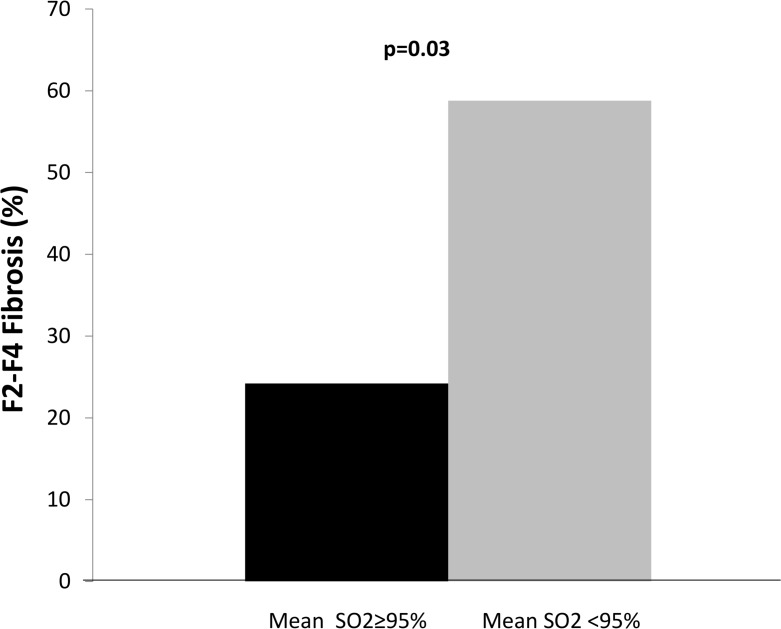
Prevalence of F2-F4 liver fibrosis among the 50 patients who underwent a sleep study during which they showed a nocturnal mean oxygen saturation < <or ≥95%.

When considering patients who underwent sleep studies, subjects with F2-F4 fibrosis, as compared to their counterparts with F0-F1 fibrosis, showed a significantly higher prevalence of AHI≥5 (72% vs 44%; p = 0.04), as well as of mean SaO2 <95% (median value in the whole group) (69% vs 38%; p = 0.03), but no significant difference in the prevalence of T90 >>1% (44% vs 24%; p = 0.12). Of note, after correction for confounders, the association between significant fibrosis and AHI***≥***5 was lost (OR 1.60, 95%C.I. 0.33–7.66; p = 0.55), while the association with mean SaO_2_ <95% was maintained (OR 3.21, 95%C.I. 1.02–7.34; p = 0.04) (Table 3). Fig 1 shows the prevalence of significant fibrosis according to mean SaO_2_ <<95%.

**Table 3:** Univariate and multivariate analyses of factors associated with F2-F4 Fibrosis in 50 patients with nonalcoholic fatty liver disease who underwent sleep study.

Variable	Unadjusted Model	Adjusted Model	Adjusted Model	Adjusted Model
**Male gender**	1.32 (0.42–4.14) 0.63	1.05 (0.23–4.78) 0.94	0.82 (0.19–3.54) 0.79	0.91 (0.21–3.93) 0.89
**Age**	1.12 (1.04–1.20) 0.001	1.14 (1.04–1.25) 0.005	1.13 (1.03–1.23) 0.005	1.13 (1.03–1.23) 0.004
**Waist Circumeference**	1.03 (0.98–1.09) 0.15	0.98 (0.91–1.06) 0.71	0.99 (0.92–1.07) 0.97	0.99 (0.92–1.07) 0.92
**HOMA**	1.33 (1.00–1.77) 0.04	1.49 (1.01–2.20) 0.04	1.39 (0.99–1.95) 0.05	1.41 (1.01–2.07) 0.04
**AHI ≥5**	3.27 (1.00–10.6) 0.04		1.60 (0.33–7.66) 0.55	
Mean Sa02 <95%*	3.61 (1.10–11.7) 0.01	3.21 (1.02–7.34) 0.04		
**T90 >1%**	2.83 (0.87–9.13) 0.08			2.43 (0.53–11.2) 0.25

Abbreviations: HOMA, homeostasis model assessment; OSA, obstructive sleep apnea; AHI, apnea-hypopnea index; SaO2, oxygen saturation; T90, percentage of total sleep time spent with SaO2<90%.

**Fig 2:**
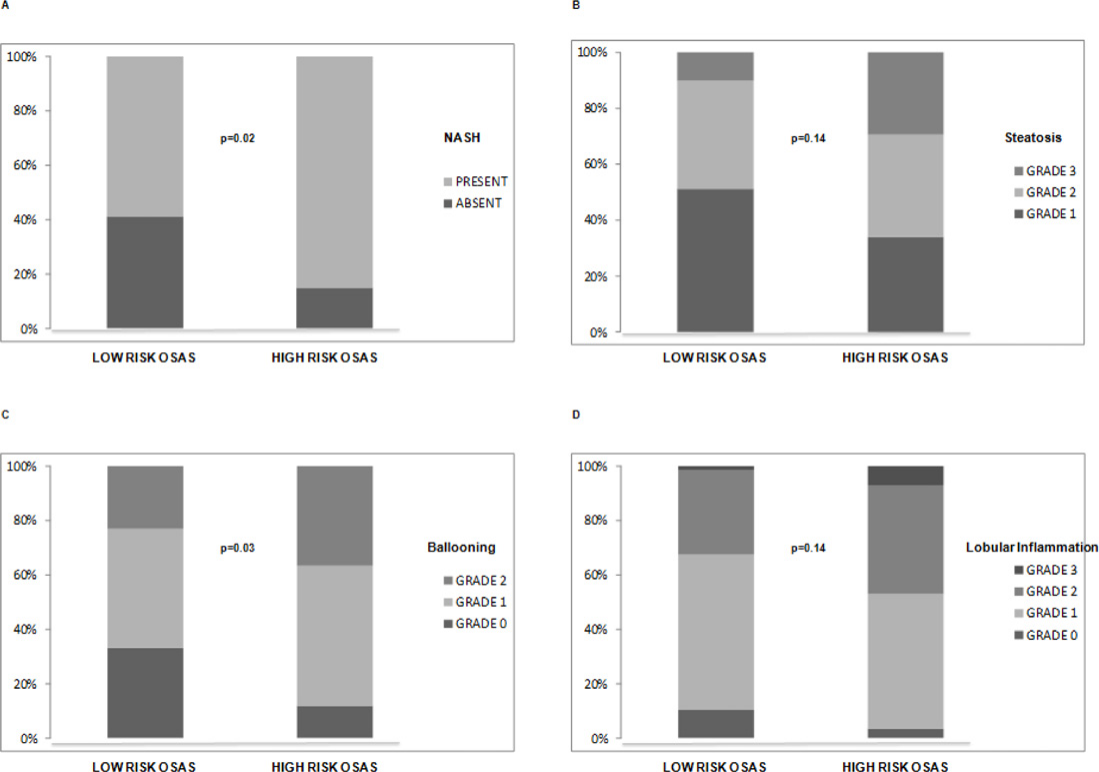
Association between NASH (A), severity of steatosis (B), severity of ballooning (C) and severity of lobular inflammation (D) with high risk OSA assessed by the STOP-BANG questionnaire. P values are adjusted for waist circumference and HOMA.

When considering the entire cohort of 126 patients, after adjusting for WC and HOMA, an independent association was observed between high risk for OSA and both NASH (p = 0.02) and ballooning (p = 0.03) (Fig 2). Similarly, in the subgroup of 50 patients who underwent sleep studies a significantly higher prevalence of NASH (88% vs 60%, p = 0.02) and ballooning (91% vs 64%; p = 0.02) was observed in those with AHI≥>5, but such associations became nonsignificant after adjusting for confounders (data not shown).

### Carotid plaques are associated with high risk for OSA and lower oxygen saturation

In the entire cohort of 126 patients the prevalence of carotid plaques was higher in patients at high risk compared to those at low risk for OSA (51.2% vs 24.3%, p = 0.006), this association being maintained after adjusting for confounders (O.R. 3.17, 95%C.I. 1.26–7.94; p = 0.01) (Table 4). Similar results were obtained when replacing WC, HOMA, hypertension and LDL-cholesterol with metabolic syndrome in the model. When IMT was assessed as a continuous variable, high risk for OSA and higher LDL-cholesterol resulted independently associated with higher IMT (p = 0.005) after adjustments for WC, HOMA, and smoking (p = 0.03).

**Table 4:** Univariate and multivariate analyses of factors associated with carotid plaques in 126 patients with nonalcoholic fatty liver disease who were administered STOP-BANG.

Variable	Unadjusted Model	Adjusted Model
**Waist Circumeference**	1.00 (0.97–1.03) 0.59	0.98 (0.95–1.02) 0.56
**HOMA**	1.19 (0.99–1.44) 0.06	1.15 (0.92–1.45) 0.20
**LDL cholesterol**	1.00 (0.99–1.01) 0.19	1.00 (0.99–1.01) 0.18
**Smoking**	1.74 (0.74–4.09) 0.19	1.73 (0.69–4.28) 0.23
**High Risk OSA**	3.26 (1.37–7.74) 0.007	3.17 (1.26–7.94) 0.01

Abbreviations: HOMA, homeostasis model assessment; LDL, low density lipoprotein; OSA, obstructive sleep apnea; AHI, apnea-hypopnea index; SaO2, oxygen saturation; T90, percentage of total sleep time spent with SaO2<90%.

**Fig 3:**
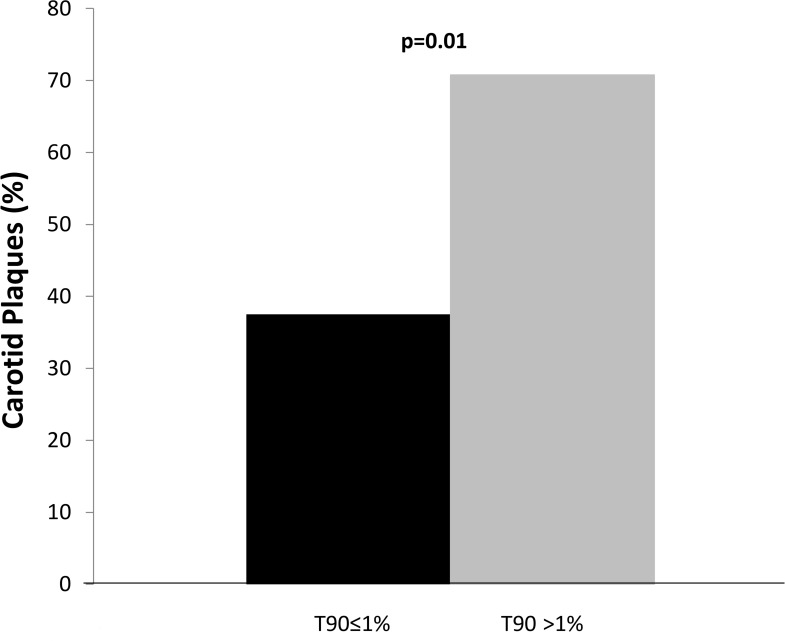
Prevalence of carotid plaques among the 50 patients who underwent a sleep study during which they spent <<1% or ≥≥1% of time with oxygen saturation ≥≥90%.

Among patients who underwent sleep studies, subjects with carotid plaques showed nonsignificant trends toward higher prevalence of OSA (64% vs 40%; p = 0.08) and of mean SaO_2_ <95% (68% vs 44%; p = 0.09), and a significantly higher occurrence of T90 >1% (52% vs 17%; p = 0.01). After correction for confounders, the association between carotid plaques and both AHI***≥***5 (OR 1.14, 95%C.I. 0.18–6.99; p = 0.88) and mean SaO_2_ <<95% (OR 0.95, 95%C.I. 0.16–5.67; p = 0.96) became nonsignificant, while the association with T90 >1% remained significant (OR 6.30, 95%C.I. 1.02–12.3; p = 0.01) (Table 5). Fig 3 shows the prevalence of carotid plaques according to T90 >1%. However, when IMT was analysed as a continuous variable, no association was found with AHI or SaO_2_ indexes (data not shown).

**Table 5:** Univariate and multivariate analyses of factors associated with carotid plaques in 50 patients with nonalcoholic fatty liver disease who underwent sleep study.

Variable	Unadjusted Model	Adjusted Model	Adjusted Model	Adjusted Model
**Male gender**	1.73 (0.54–5.53) 0.35	1.06 (0.23–4.95) 0.93	0.81 (0.18–3.57) 0.78	0.81 (0.18–3.60) 0.78
**Age**	1.13 (1.04–1.22) 0.002	1.12 (1.02–1.23) 0.01	1.13 (1.03–1.24) 0.008	1.13 (1.03–1.24) 0.008
**Waist Circumeference**	1.03 (0.98–1.09) 0.18	1.03 (0.95–1.11) 0.40	1.04 (0.96–1.12) 0.32	1.04 (0.96–1.12) 0.31
**HOMA**	1.13 (1.88–1.44) 0.33	1.17 (0.84–1.63) 0.33	1.08 (0.79–1.45) 0.61	1.08 (0.80–1.46) 0.60
**LDL cholesterol**	1.00 (0.99–1.02) 0.39	1.02 (0.99–1.04) 0.07	1.01 (0.99–1.04) 0.10	1.01 (0.99–1.04) 0.10
**Arterial Hypertension**	2.81 (0.87–9.09) 0.08	0.73 (0.12–4.42) 0.73	1.20 (0.18–7.86) 0.84	1.28 (0.24–6.76) 0.76
**Smoking**	1.13 (0.29–4.38) 0.85	0.72 (0.09–5.41) 0.75	1.58 (0.27–9.15) 0.60	1.58 (0.25–9.79) 0.62
**AHI ≥5**	2.81 (0.87–9.09) 0.08		1.14 (0.18–6.99) 0.88	
**Mean Sa02 <95%**	2.76 (0.85–8.96) 0.09			0.95 (0.16–5.67) 0.96
**T90 >1%**	4.58 (1.38–15.1) 0.01	6.30 (1.02–12.3) 0.01		

Abbreviations: HOMA, homeostasis model assessment; LDL, low density lipoprotein; OSA, obstructive sleep apnea; AHI, apnea-hypopnea index; SaO2, oxygen saturation; T90, percentage of total sleep time spent with SaO2<90%.

## Discussion

In a western cohort of biopsy-proven NAFLD patients with a low prevalence of morbid obesity and a high prevalence of significant liver fibrosis, 69% of patients were at high risk for OSA according to the STOP-BANG questionnaire. Among patients undergoing a nocturnal respiratory polygraphy, an AHI ≥5 at was present in half of the cases, and some indexes of oxygen saturation resulted independently associated with both the severity of liver fibrosis and carotid atherosclerosis. Of note, these associations were maintained after correction for both hepatic and cardiometabolic confounders despite potentially low statistical power due to the small sample size.

In the last years, several studies have reported a strong link between OSA and cardiometabolic alterations, as well as between OSA and direct, i.e., hepatic histology—or indirect, i.e., ALT or GGT levels, markers of liver damage in NAFLD patients [13–29]. In our cohort we observed an independent association between severity of liver damage, namely significant fibrosis, NASH and ballooning, and high risk for OSA assessed by the STOP-BANG questionnaire. Our results agree with those reported by Pulixi and colleagues [28] who studied a similar cohort of biopsy-proven adult NAFLD patients without morbid obesity, but used a different tool for OSA screening, i.e. the Berlin questionnaire. Instead, in a similar clinical setting no association between high risk for OSA and liver damage was reported by Singh and colleagues [27]; however, in that study liver damage was estimated based on serum ALT, which is a less reliable surrogate of liver damage [44].

In our study, prevalence of AHI≥5 was 50% according to cardiorespiratory polygraphy. To the best of our knowledge, this is the first study showing an independent association between nocturnal oxygen saturation values and significant liver fibrosis in adult patients with biopsy-proven, severe NAFLD at low prevalence of morbid obesity. Similar observations were previously obtained in cohorts of bariatric patients [15–22], who differ for anthropometric and perhaps genetic features from “normal” NAFLD, and in two studies on children with NAFLD [25,26].

NAFLD patients are known to be at high risk of vascular dysfunction and carotid atherosclerosis due to the high prevalence of metabolic comorbidities and to their pro-atherogenic and pro-coagulant imbalance [33]. In our sample of NAFLD patients we found that a high risk for OSA as well as a T90 >1% were independently associated with subclinical carotid atherosclerosis evaluated as IMT and carotid plaques. Therefore, our data suggest that OSA could contribute to atherogenic lesions in NAFLD patients, possibly through the effects of nocturnal exposure to hypoxia. Many studies have reported a pathogenetic link between OSA and atherosclerosis, albeit with some disagreement [13]. Specifically, studies performed in different settings like general population or patients at risk for OSA highlighted that OSA and/or oxygen saturation indexes are associated with IMT and/or carotid plaques [30–32]. Our study however is the first one reporting these associations in a setting of NAFLD patients at low prevalence of morbid obesity.

From a clinical point of view, our study suggests that occurrence of sleep disordered breathing in NAFLD patients should be assessed by following a 2-step approach: first, administration of the STOP-BANG questionnaire and then, in patients positive to the questionnaire, a nocturnal sleep study. In fact, the questionnaire proved highly sensitive but poorly specific in our patients, so that occurrence of OSA could be overestimated by using only that tool. Moreover, the symptom of daytime sleepiness was rarely reported by NAFLD patients. The identification of OSA in patients with NAFLD could be followed by OSA treatment, with benefits on metabolic and perhaps liver alterations. A recent longitudinal study from our group suggested that OSA treatment by continuous positive airway pressure might be associated with decreased liver steatosis [45].

Our study is merely observational and was not designed to explore mechanisms for the association of OSA and either liver fibrosis or atherosclerosis in NAFLD patients. However, according to recent evidence, we may put forward a few hypotheses to be further tested in experimental studies. Intermittent hypoxia, in addition to systemic inflammation, could promote atherosclerosis and liver damage as observed in murine models of NAFLD [46–49], and liver profibrogenic infiltrate as expressed by increased intrahepatic leukocytes and activated macrophages/Kupffer cells in human NAFLD [24,25]. In addition, the interplay between decreased oxygen saturation, liver damage, vascular alterations and metabolic dysfunction can stem from a common pathway that hypoxia and insulin resistance can activate and amplify. Experimental studies showed that both insulin resistance and intermittent hypoxia are able to stimulate the carotid bodies, i.e. peripheral chemoreceptors that classically respond to hypoxia causing hyperventilation through an increase in the carotid sinus nerve activity In addition, the carotid sinus nerve discharge increases the activity of the sympathoadrenal system, and amplifies mechanisms leading to insulin resistance and its systemic complications, including liver and vascular damage [50,51]. However, our data, while suggesting a possible role for nocturnal hypoxia in such alterations, do not indicate a specific and/or exclusive role of intermittent hypoxia. Both mean SaO_2_ and T90 reflect the nocturnal hypoxic load, rather than the intermittent cycles of hypoxia/reoxygenation associated with respiratory events. Nobili and coworkers also found that T90 was the strongest predictor of NASH severity and fibrosis stage in pediatric NAFLD (25). Finally, as demonstrated in children with NAFLD, OSA could induce liver damage by increasing intestinal permeability and endotoxemia, that in turn activate Kupffer and hepatic stellate cells, and by expanding adiponectin-deficient hepatic progenitor cells, key features of steatohepatitis and fibrosis [52]. Further studies are needed to better understand the role of nocturnal hypoxia as a marker of increased risk in NAFLD, and to assess the potential usefulness of nocturnal pulse oximetry studies in patients affected by this disease.

The study has both strengths and limitations. The main strength is the availability of a homogeneous cohort of biopsy-proven NAFLD patients, with a low prevalence of morbid obesity, fully studied for sleep disordered breathing, as well as for metabolic, liver and carotid alterations. However, results were obtained in a cohort of Italian patients with elevated ALT levels and a high prevalence of NASH and F2-F4 fibrosis enrolled at a tertiary care center, and may not be readily applicable to patients with NAFLD from the general population. The cross-sectional nature of our study does not prove a causal link between OSA and liver or vascular damage. A further methodological issue is the relative small sample size. Besides, more than 50% of the studied population refused the sleep study. Nevertheless, prevalence of high risk for OSA, based on the results of the questionnaire, was similar in patients with and without sleep studies, even if the former ones had a higher prevalence of metabolic comorbidities. Although hypoxemia could have been also related to a cardiac cause, the lack of cardiological symptoms, the exclusion of patients with a previous history of cardiovascular disorders, and the fact that a high proportion of these patients underwent echocardiography without evidence of severe cardiac alterations [53] make this possibility unlikely. Finally, OSA was not diagnosed by full polysomnography, but our choice to use cardiorespiratory polygraphy was warranted by the widespread and increasing acceptance of the reliability of this diagnostic tool [54].

In conclusion, in a cohort of NAFLD patients enrolled in a tertiary care center and showing a low prevalence of morbid obesity, AHI≥5 was highly prevalent. Our data suggest that indexes of oxygen saturation were associated with severity of liver damage and of carotid atherosclerosis risk. These data, if further validated, suggest that occurrence of sleep disordered breathing should be investigated in NAFLD patients, since OSA is a treatable condition and may represent a potential additional therapeutic target for NAFLD.

## Supporting Information

S1 Table Baseline Demographic, Laboratory, Metabolic, and Histological Features of 126 Italian Patients with biopsy-proven Non-alcoholic Fatty Liver Disease, according to acceptance or refusal of sleep study. (DOCX)

S2 Table Baseline Demographic, Laboratory, and Metabolic Features of 126 Italian Patients with biopsy-proven Non-alcoholic Fatty Liver Disease, according to high or low risk for obstructive sleep apnea assessed with the STOP-BANG questionnaire. (DOCX)

## Abbreviations

IR: insulin resistance
NAFLD: non-alcoholic fatty liver disease
NASH: non-alcoholic steatohepatitis
HOMA: homeostasis model assessment
OSA: Obstructive Sleep Apnea Syndrome
