# Supplementary material for: Obstructive Sleep Apnea Is Associated with Liver Damage and Atherosclerosis in Patients with Non-Alcoholic Fatty Liver Disease
Source: PLoS One. 2015 Dec 16;10(12):e0142210. doi: 10.1371/journal.pone.0142210 (PMC4682677; doi:10.1371/journal.pone.0142210)
Supplement: S1 Table — (DOCX) [file pone.0142210.s001.docx]

**S1 Table. Baseline Demographic, Laboratory, Metabolic, and Histological Features of 126 Italian Patients with biopsy-proven Non-alcoholic Fatty Liver Disease, according to acceptance or refusal of sleep study.**

| **Variable** | **Patients who refused sleep study**  n=76 | **Patients who underwent**  **sleep study**  n=50 | **P value** |
| --- | --- | --- | --- |
| **Age** – **years** | 48.8 ± 12.9 | 52.9 ± 10.9 | 0.07 |
| **Male Gender - % of subjects** | 69 | 58 | 0.17 |
| **BMI - kg/m^2^**  **BMI<25 kg/m^2^ - % of subjects**  **BMI≥25-<30 kg/m^2^- % of subjects**  **BMI≥30-<35 kg/m^2^- % of subjects**  **BMI≥35-<40 kg/m^2^- % of subjects**  **BMI≥40 kg/m^2^- % of subjects** | 28.9 ± 4.8  19.7  46.0  25.0  5.4  3.9 | 32.2 ± 4.7  8.0  30.0  36.0  20.0  6.0 | 0.01  0.01 |
| **Waist Circumference – cm**  **Visceral Obesity - % of subjects** | 102.3 ± 11.9  63 | 106.6 ± 11.4  80 | 0.04  0.04 |
| **Alanine Aminotransferase – IU/ml** | 79.6 ± 50.8 | 65.0 ± 36.6 | 0.08 |
| **Blood glucose – mg/dl** | 95.1 ± 25.3 | 108.0 ± 32.7 | 0.01 |
| **Insulin – IU** | 14.2 ± 7.1 | 18.1 ± 10.3 | 0.03 |
| **HOMA** | 3.43 ± 1.57 | 4.43 ± 2.48 | 0.003 |
| **Type 2 Diabetes - % of subjects** | 14 | 40 | 0.001 |
| **Arterial Hypertension - % of subjects** | 23 | 50 | 0.002 |
| **Metabolic Syndrome - % of subjects** | 24 | 40 | 0.02 |
| **Smoking - % of subjects** | 22 | 22 | 0.96 |
| **Cholesterol – mg/dl** | 202.6 ± 46.2 | 199.6 ± 44.8 | 0.72 |
| **HDL Cholesterol – mg/dl** | 49.9 ± 13.6 | 49.1 ± 14.3 | 0.19 |
| **LDL Cholesterol – mg/dl** | 124.9 ± 39.0 | 119.2 ± 37.5 | 0.42 |
| **Triglycerides – mg/dl** | 136.4 ± 75.2 | 130.1 ± 67.7 | 0.64 |
| **Intima Media Thickness - mm** | 0.82 ± 0.24 | 0.82 ± 0.16 | 0.86 |
| **Carotid Plaque - % of subjects** | 37.5 | 50 | 0.10 |
| **High Risk for OSA (STOP-BANG >2) - % of subjects** | 68 | 76 | 0.17 |
| **Tiredness (STOP-BANG) - % of subjects** | 53.9 | 66 | 0.18 |
| **Histology** |  |  |  |
| **Lobular inflammation 2-3** **- % of subjects** | 40 | 42 | 0.74 |
| **Steatosis grade 3 (>66%) - % of subjects** | 21 | 26 | 0.67 |
| **Ballooning - % of subjects** | 82 | 74 | 0.42 |
| **NASH - % of subjects** | 79 | 74 | 0.51 |
| **Fibrosis Stage 2-4 - % of subjects** | 43 | 58 | 0.10 |

Abbreviations: IU, international units; HOMA, homeostasis model assessment; HDL, high density lipoprotein; LDL, low density lipoprotein; AHI, apnea-hypopnea index; SaO2, oxygen saturation; T90%, percentage of total sleep time spent with SaO2<90; ESS, Epworth Sleepiness Scale. Data are given as mean ± standard deviation, or as median (95% C.I.), or as %.
