# Supplementary material for: Obstructive Sleep Apnea Is Associated with Liver Damage and Atherosclerosis in Patients with Non-Alcoholic Fatty Liver Disease
Source: PLoS One. 2015 Dec 16;10(12):e0142210. doi: 10.1371/journal.pone.0142210 (PMC4682677; doi:10.1371/journal.pone.0142210)
Supplement: S2 Table — (DOCX) [file pone.0142210.s002.docx]

**S2 Table. Baseline Demographic, Laboratory, and Metabolic Features of 126 Italian Patients with biopsy-proven Non-alcoholic Fatty Liver Disease, according to high or low risk for obstructive sleep apnea assessed with the STOP-BANG questionnaire.**

| **Variable** | **Low Risk for OSA**  n=39 | **High Risk for OSA**  n=87 | **P value** |
| --- | --- | --- | --- |
| **Age** – **years** | 41.3 ± 10.3 | 54.2 ± 11.1 | <0.001 |
| **Male Gender - % of subjects** | 59 | 68 | 0.33 |
| **BMI Kg/m**  **BMI<25 Kg/m^2^- % of subjects**  **BMI≥25-<30 Kg/m^2^- % of subjects**  **BMI≥30-<35 Kg/m^2^- % of subjects**  **BMI≥35-<40 Kg/m^2^- % of subjects**  **BMI≥40 Kg/m^2^- % of subjects** | 28.2 ± 4.8  25.6  38.4  28.3  5.2  2.5 | 30.6 ± 4.8  10.3  40.2  29.9  13.8  5.8 | 0.01  0.01 |
| **Waist Circumference – cm**  **Visceral Obesity - % of subjects** | 100.1 ± 10.1  59 | 105.8 ± 12.2  75 | 0.01  0.07 |
| **Alanine Aminotransferase – IU/ml** | 88.6 ± 62.1 | 67.2 ± 33.5 | 0.01 |
| **Blood glucose – mg/dl** | 96.3 ± 33.8 | 102.0 ± 26.7 | 0.30 |
| **Insulin – IU** | 12.6 ± 7.1 | 17.0 ± 8.9 | 0.01 |
| **HOMA** | 3.07 ± 1.67 | 4.09 ± 2.13 | 0.009 |
| **Type 2 Diabetes - % of subjects** | 8 | 32 | 0.003 |
| **Arterial Hypertension - % of subjects** | 5 | 47 | <0.001 |
| **Metabolic Syndrome - % of subjects** | 18 | 33 | 0.07 |
| **Smoking - % of subjects** | 15 | 25 | 0.21 |
| **Cholesterol – mg/dl** | 203.8 ± 45.1 | 200.3 ± 45.8 | 0.69 |
| **HDL Cholesterol – mg/dl** | 51.6 ± 13.4 | 51.6 ± 19.7 | 0.99 |
| **LDL Cholesterol – mg/dl** | 128.6 ± 37.6 | 119.5 ± 38.4 | 0.22 |
| **Triglycerides – mg/dl** | 117.5 ± 70.0 | 141.7 ± 71.8 | 0.08 |
| **Intima Media Thickness – mm** | 0.74 ± 0.18 | 0.86 ± 0.21 | 0.005 |
| **Carotid Plaque - % of subjects** | 24% | 51% | 0.006 |
| **AHI* - no/hour** | 0.9 (0.1-4.7) | 6.7 (1.3-51.9) | <0.001 |
| **AHI ≥5* - % of subjects**  **Basal Sa02* - %** | 0  97.0±0.8 | 66  95.7±1.3 | <0.001  0.003 |
| **Mean Sa02* - %** | 95.2±1.2 | 93.4±2.0 | 0.006 |
| **Mean Sa02 <95%* - % of subjects** | 8 | 71 | <0.001 |
| **T90 * - %** | 0 (0-0.2) | 0.7 (0-57.2) | 0.001 |
| **T90 >1%* - % of subjects** | 8 | 47 | 0.01 |
| **ESS score*** | 6 (2-12) | 7 (2-13) | 0.08 |
| **ESS ≥10 (%)*- % of subjects** | 16 | 16 | 0.89 |

Abbreviations: IU, international units; HOMA, homeostasis model assessment; HDL, high density lipoprotein; LDL, low density lipoprotein; AHI, apnea-hypopnea index; SaO2, oxygen saturation; T90, percentage of total sleep time spent with SaO2<90; ESS, Epworth Sleepiness Scale. Data are given as mean ± standard deviation, or as median (95% C.I.), or as %.

*Data available for 12 patients in the group with low risk for obstructive sleep apnea, and for 38 patients in the group with high risk for obstructive sleep apnea.
